# Premenopausal bilateral oophorectomy and Alzheimer's disease imaging biomarkers later in life

**DOI:** 10.1002/alz.14469

**Published:** 2024-12-23

**Authors:** Kejal Kantarci, Ekta Kapoor, Jennifer R. Geske, Anna Castillo, Julie A. Fields, Firat Kara, Evdokiya E. Knyazhanskaya, Christopher G. Schwarz, Matthew L. Senjem, Kent R. Bailey, Val Lowe, Nathan K. LeBrasseur, Walter A. Rocca, Michelle M. Mielke

**Affiliations:** ^1^ Department of Radiology Division of Neuroradiology Mayo Clinic Rochester Minnesota USA; ^2^ Mayo Clinic Women's Health Research Center Mayo Clinic Rochester Minnesota USA; ^3^ Department of Internal Medicine Division of Endocrinology Mayo Clinic Rochester Minnesota USA; ^4^ Department of Quantitative Health Sciences Mayo Clinic Rochester Minnesota USA; ^5^ Department of Psychiatry and Psychology Mayo Clinic Rochester Minnesota USA; ^6^ Mayo Clinic Alix School of Medicine Mayo Clinic Rochester Minnesota USA; ^7^ Department of Information Technology Mayo Clinic Rochester Minnesota USA; ^8^ Department of Physical Medicine and Rehabilitation Mayo Clinic Rochester Minnesota USA; ^9^ Division of Epidemiology Department of Quantitative Health Sciences Mayo Clinic Rochester Minnesota USA; ^10^ Department of Neurology Mayo Clinic Rochester Minnesota USA; ^11^ Department of Epidemiology and Prevention Wake Forest University School of Medicine Winston‐Salem North Carolina USA

**Keywords:** amyloid, atrophy, menopause, magnetic resonance imaging, oophorectomy, positron emission tomography, premenopausal oophorectomy, tau

## Abstract

**INTRODUCTION:**

Premenopausal bilateral oophorectomy (PBO) before the age of 46 years is associated with an increased risk of dementia. We investigated the long‐term effects of PBO performed before age 50 years on amyloid beta (Aβ), tau, and neurodegeneration imaging biomarkers of Alzheimer's disease (AD).

**METHODS:**

Mayo Clinic Cohort Study of Oophorectomy and Aging‐2 participants were divided into early PBO (< 46 years; *n* = 61), and late PBO (46–49 years; *n* = 51) groups and were compared to referent women who did not undergo PBO (*n* = 119).

**RESULTS:**

Early PBO was associated with thinner entorhinal cortex (*p* = 0.014), higher tau load at higher levels of Aβ load (*Pp* = 0.005), higher Aβ load (*p* = 0.026), and smaller temporal lobe cortical thickness (*p* = 0.022), only at older ages compared to the referent group.

**DISCUSSION:**

PBO before the age of 46 years is associated with entorhinal cortex thinning, elevated tau at higher Aβ levels, along with an AD‐like pattern of atrophy at older ages.

**CLINICAL TRIALS REGISTRATION:**

NCT03821857 sex‐specific effects of endocrine disruption on aging and AD.

**Highlights:**

- Premenopausal bilateral oophorectomy (PBO) before the ages of 46 (early PBO) years and ages 46 to 49 (late PBO) years was studied.
- Early PBO was associated with reduced entorhinal cortex thickness later in life.
- Early PBO was associated with greater amyloid beta (Aβ) load at older ages.
- Early PBO was associated with greater Alzheimer's disease pattern of atrophy at older ages.
- Early PBO was associated with higher tau load at higher Aβ levels.

## 1 BACKGROUND

The social and economic burden of dementia in the coming decades will be greatest in women because of their longer life expectancy and resulting elevated lifetime risk for dementia compared to men.^1^ This elevated risk of dementia in women may be, in part, modulated by ovarian hormones^2^ and by the apolipoprotein E (*APOE*) ε4 genotype.^3^, ^4^, ^5^ Women who undergo premenopausal bilateral oophorectomy (PBO) particularly before the age of 46 years have an accelerated accumulation of multimorbidity^6^ and an increased risk of mortality due to aging‐related neurological diseases including dementia,^7^ the most common type being Alzheimer's disease (AD). Determining the effects of PBO on the risk of AD dementia would require decades of follow‐up; alternatively, imaging biomarkers can be used for assessing the effects of an abrupt loss of ovarian hormones on the risk of AD in a shorter time frame. Furthermore, imaging biomarkers may provide insight into the underlying pathogenic mechanisms of cognitive impairment and dementia associated with PBO.

In the revised criteria for the diagnosis and staging of AD, positron emission tomography (PET) imaging of amyloid beta (Aβ) and neurofibrillary tangle tau pathologies are considered the essential biomarkers of AD neuropathologic changes (i.e., Core 1 biomarkers).^8^ Neurodegeneration, measured with the AD‐like pattern of cortical atrophy, and entorhinal cortex thinning on magnetic resonance imaging (MRI) are less specific to AD, but could characterize the early neurodegenerative changes associated with the AD pathology.^8^, ^9^ The imaging biomarkers of AD are being used for early diagnosis and staging of AD pathology even in cognitively unimpaired individuals decades before they develop cognitive impairment and dementia.^10^ Furthermore, Aβ and neurofibrillary tangle tau biomarkers of AD are being used to identify patients for prevention trials of disease‐modifying therapies before clinical symptoms are detected.^11^, ^12^ Hence, the AD imaging biomarkers can elucidate the association of PBO with the pathophysiology of AD later in life.

In the current study, we investigated the long‐term effects of the abrupt ovarian hormonal disruption caused by PBO on imaging biomarkers of AD in a cohort of women who underwent PBO before the age of 50 years compared to age‐matched referent women. Based on the literature indicating that PBO before age 46 years increases the risk of cognitive impairment and dementia later in life, we hypothesized that the imaging biomarkers of AD would be abnormal in women who underwent PBO before the age of 46 years (premature or early PBO), but not in women who underwent PBO at ages of 46 to 49 years (late PBO) compared to referent women who did not undergo PBO. We further examined whether age at assessment with imaging or *APOE* ε4 status modified these differences.

## 2 METHODS

### 2.1 Participants

**FIGURE 1 alz14469-fig-0001:**
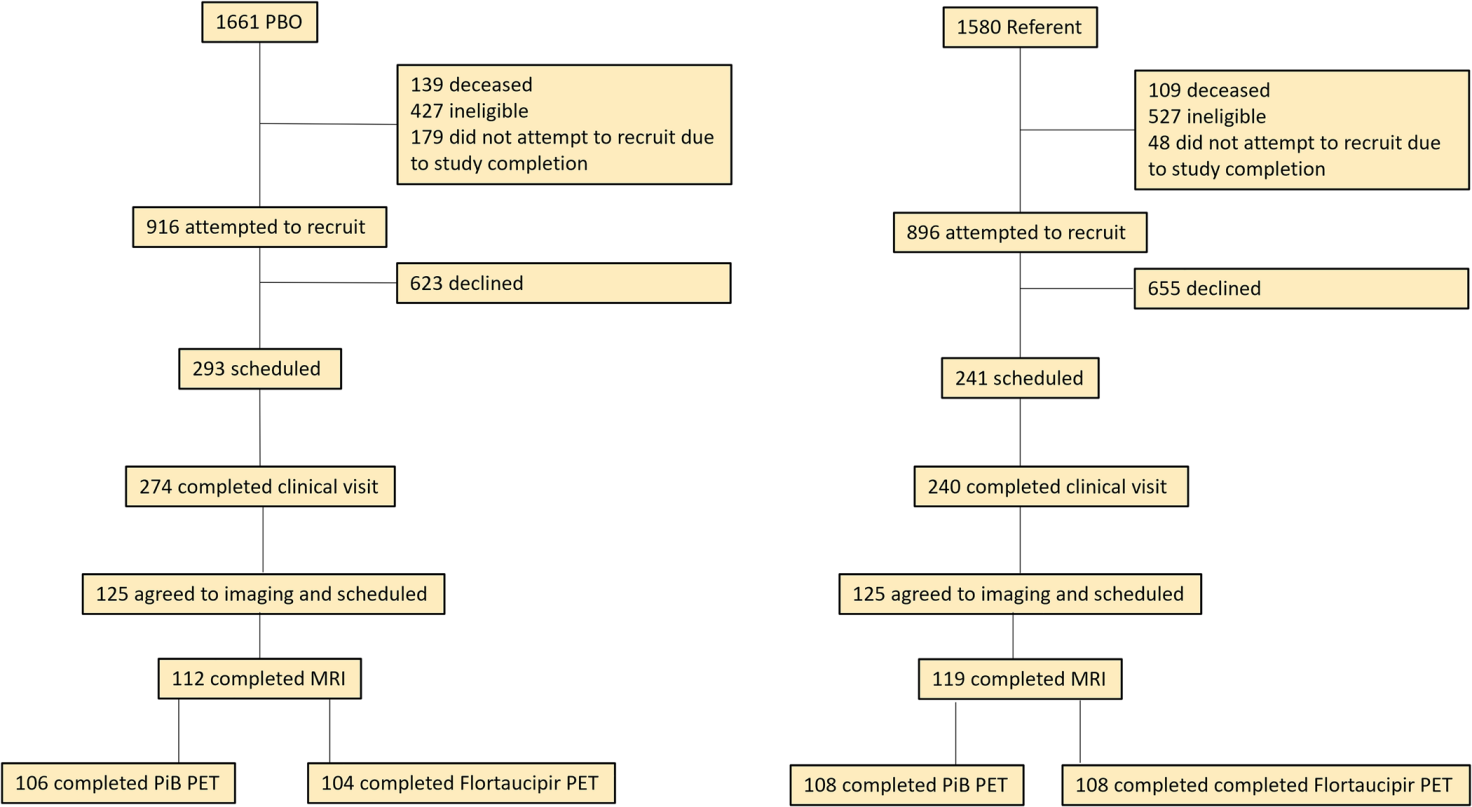
Recruitment flowchart: among the MOA‐2 participants who underwent PBO (*n* = 1661), 112 women fulfilled the inclusion and exclusion criteria and participated in the current study. Among the age‐matched MOA‐2 participants who did not undergo PBO (i.e., referent women; *n* = 1580), 119 women fulfilled the inclusion and exclusion criteria and participated in the current study. MOA‐2, Mayo Clinic Cohort Study of Oophorectomy and Aging‐2; MRI, magnetic resonance imaging; PET, positron emission tomography; PiB, Pittsburgh compound B; PBO, premenopausal bilateral oophorectomy.

Participants of the current study were recruited from the Mayo Clinic Cohort Study of Oophorectomy and Aging‐2 (MOA‐2). MOA‐2 included a cohort of women who underwent PBO before age 50 years and before reaching spontaneous menopause from 1988 through 2007, and a cohort of age‐matched referent women who had not undergone PBO before the index date (date of matching). All women resided in a geographically defined population of the Rochester Epidemiology Project (REP) medical records–linkage system. PBO was defined as complete removal of both ovaries or as second unilateral oophorectomy, with or without hysterectomy, and performed before the onset of spontaneous menopause. Women were excluded if they underwent PBO: (1) before the age of 10 years, (2) for ovarian cancer (primary or metastatic), (3) for the treatment of another estrogen‐sensitive malignancy (usually breast cancer), or (4) for a high risk of ovarian cancer as judged by the gynecologist based on family history or confirmed by genetic testing.^13^ The recruitment procedures for the current study are detailed elsewhere.^14^ Briefly, a randomized list of all women who underwent PBO (*n* = 916) and age‐matched referent women (*n* = 896) was created from the MOA‐2 cohort and these potential participants were contacted in a sequential manner. Women were eligible if they were: (1) identified and passively followed in MOA‐2, (2) aged ≥ 60 years at enrollment, (3) > 6 months post chemotherapy or major surgery requiring general anesthesia, and (4) willing and able to sign the informed consent. Women were ineligible if they were: (1) not able to read or speak English, (2) in hospice, (3) had contraindications for MRI such as claustrophobia or MRI‐incompatible implants, (4) did not have a permanent residence within ≈ 200 miles from Rochester, Minnesota (due to the need for in‐person clinic visits), or (5) had not had a clinical visit in the REP in the past 5 years. Women were recruited from 2018 through 2022, when the study was completed (Figure 1). This study was approved by the Mayo Clinic and Olmsted Medical Center Institutional Review Boards and informed consent was obtained from all participants.

### 2.2 MRI and PET acquisition

Participants underwent brain MRI on a Siemens Prisma scanner (V11e software) with 64 channel receiver head coil. Magnetization prepared rapid gradient echo (MPRAGE) sequence with a 0.8 mm isotropic resolution was acquired. PET images were acquired using a PET/computed tomography scanner (D690; GE Healthcare). PET scans were performed with the injection of an average of 386 MBq (range, 347–406 MBq) flortaucipir, followed by an 80 minute uptake period. In another scan session, an average of 596 MBq (range, 292–729 MBq) 11C‐Pittsburgh compound B (PiB) was injected followed by a 40 minute uptake period, and a 20 minute PiB PET scan consisting of four 5 minute dynamic frames was obtained. For each woman, imaging studies were completed within a period of 3 months.

**RESEARCH IN CONTEXT**

1. **Systematic review**: PubMed was used to review the literature. Although the imaging biomarkers of Alzheimer's disease (AD) have not been studied specifically in women with premenopausal PBO, there are several publications on multimorbidity, survival, and cognitive outcomes of premenopausal bilateral oophorectomy (PBO). These were appropriately cited.
2. **Interpretation**: Our findings indicate that PBO before the age of 46 years is associated with abnormalities in imaging biomarkers of AD later in life. These findings were not present in women who underwent PBO at the age of 46 to 49 years.
3. **Future directions**: Women who underwent PBO before the age of 46 years had elevated amyloid beta (Aβ) only at older ages and elevated tau at higher Aβ levels compared to women who did not undergo PBO. Longitudinal follow‐up with serial imaging of women who underwent PBO before the age of 46 years may reveal the evolution of AD in these women, which is critical for preventive strategies.

### 2.3 MRI and PET analysis

MPRAGE MRI were tissue‐class segmented and corrected for B0 inhomogeneities using Unified Segmentation^15^ in SPM12 (www.fil.ion.ucl.ac.uk/spm) with population‐optimized templates and settings from the Mayo Clinic Adult Lifespan Template (MCALT; https://www.nitrc.org/projects/mcalt/). Tissue volumes were calculated by summing voxel‐wise probabilities using ANTS Symmetric Normalization propagated from anatomically labeled atlas regions from the MRI MCALT template,^16^ with right and left hemispheric values averaged as previously described.^17^ We then estimated cortical thickness from these segmentations using registration‐based cortical thickness^18^ within the regions of AD‐pattern neurodegeneration. These regions included the entorhinal cortex as the earliest region to be involved in AD‐related neurodegeneration on MRI,^19^ and a temporal lobe meta‐region of interest that included entorhinal, fusiform, parahippocampal, midtemporal, inferior temporal, and angular gyrus cortical regions as previously described.^17^

Quantitative image analysis for PET was performed using an in‐house fully automated image processing pipeline as previously described.^20^ Briefly, each PET image was rigidly registered to its corresponding MPRAGE using SPM12, and regional PET values were extracted from anatomically labeled atlas regions propagated from the MCALT template. A composite cortical PiB standardized uptake volume ratio (SUVR) was calculated by taking the median uptake in prefrontal, orbitofrontal, parietal, temporal, anterior cingulate, and posterior cingulate/precuneus regions, and normalizing by the median Aβ PET uptake in the cerebellar crus gray matter. Similarly, a composite flortaucipir SUVR was calculated by taking the median flortaucipir uptake in the entorhinal, amygdala, parahippocampal, fusiform, inferior temporal, and middle temporal regions and normalizing by the median flortaucipir uptake in the cerebellar crus gray matter.

### 2.4 Statistical analysis

Descriptive statistics for[Fig alz14469-fig-0001] demographic and clinical characteristics are provided for referent, early PBO (< 46 years), and late PBO (46–49 years) groups. Statistical comparisons of the early PBO versus referent, and late PBO versus referent groups were conducted using two‐sample *t* tests or Wilcoxon rank tests for continuous variables, and chi‐square tests or Fisher exact tests for categorical variables. Age‐adjusted linear regression models were used to compare body mass index (BMI) and Modified Mini‐Mental State Examinations (3MS) for those same groups. MRI and PET measures were analyzed using multivariable linear regression, stratified by early PBO (< 46 years) and late PBO (46–49 years) groups. Models included covariates to adjust for age at imaging and *APOE* ε4 carrier status. Log10 transformation of global cortical PiB SUVR values (Aβ load) was applied to create a more normal distribution. Analyses were reported with and without interactions of PBO status with age at imaging to test for an age‐dependent effect of PBO as well as for an overall effect of PBO, adjusted for age. In addition, an interaction between *APOE* ε4 carrier status and PBO was assessed. Interactions of PBO status with age at imaging and *APOE* ε4 were used to test for an age‐ and *APOE* ε4‐dependent effect of PBO. We further explored the interaction of PBO status with PiB SUVR for differences in flortaucipir SUVR (tau load) in early and late PBO groups compared to the referent group. Finally, imaging variables were correlated with the 3MS scores in each group using multivariable linear regression models adjusted by age or by both age and *APOE* ε4 status. Analyses were conducted using SAS (version 9.4).

## 3 RESULTS

Among the MOA‐2 participants who underwent PBO (*n* = 916), 112 PBO (61 early PBO and 51 late PBO) women fulfilled the inclusion and exclusion criteria and participated in the current imaging study. Among the age‐matched MOA‐2 participants who did not undergo PBO before the index date (i.e., referent women; *n* = 896), 119 women fulfilled the inclusion and exclusion criteria and participated in the current imaging study (Figure 1). The median (interquartile range) time from PBO to the current study visit was 23.4 (20.1, 27.3) years for the early PBO group and 19.7 (16.7, 24.8) years for the late PBO group. Table 1 shows the demographic and clinical characteristics of the early PBO, late PBO, and referent groups. The early PBO group was younger than the referent group at the time of assessments; therefore, all analyses comparing the PBO groups to the referent group were adjusted by age. Cognitive performance measured with the 3MS was not different in early PBO and late PBO groups compared to the referent group. The frequency of hormone therapy (HT) at age 50 years was 65.6% in the early PBO and 77.1% in late PBO groups. The frequency of diagnosis of mild cognitive impairment or dementia was not statistically significantly different when early PBO (18.0%) and late PBO (19.6%) groups were compared to the referent group (15.6%).

**TABLE 1:** Participant characteristics at the time of imaging assessment.

	Referent	Early PBO (< 46 years)	Late PBO (≥ 46 years)	Early PBO versus referent	Late PBO versus referent
**Characteristics**	(*N* = 119)	(*N* = 61)	(*N* = 51)	*p* value	*p* value
Current visit age (years)	67 (63, 70)	64 (62, 68)	67 (64, 72)	**0.003**	0.23
Modified Mini‐Mental State Examination^a^	97 (94, 99)	96 (93,99)	96 (94, 98)	0.36	0.46
Body mass index (kg/m^2^)^a^	29.0 (24.7, 33.7)	28.4 (25.4, 32.6)	30.3 (27.2, 35.3)	0.81	0.14
*APOE* ε4 carrier, *n* (%)	27 (22.9%)	14 (23.7%)	11 (22.0%)	0.90	0.90
Current marital status, *n* (%)^b^				0.65	0.43
Never married	4 (3.4%)	2 (3.3%)	1 (2.0%)		
Separated/divorced	22 (18.5%)	9 (14.8%)	4 (7.8%)		
Widowed	9 (7.6%)	5 (8.2%)	7 (13.7%)		
Married	83 (69.7%)	43 (70.5%)	37 (72.5%)		
Living as married	1 (0.8%)	2 (3.3%)	2 (3.9%)		
Education, *n* (%)^c^				0.74	0.98
High school or less	20 (17.0%)	13 (21.3%)	9 (18.8%)		
Some college	48 (40.7%)	24 (39.3%)	20 (41.7%)		
4‐year college degree	28 (23.7%)	16 (26.2%)	10 (20.8%)		
Graduate or professional degree	22 (18.6%)	8 (13.1%)	9 (18.8%)		
Raced				0.78	0.76
White	108 (91.5%)	57 (95.0%)	45 (93.8%)		
Black	0 (0%)	0 (0%)	2 (4.2%)		
Asian	2 (1.7%)	0 (0%)	0 (0%)		
Other	8 (6.8%)	3 (5.0%)	1 (2.1%)		

*Note*: Statistical comparisons of the early PBO versus referent, and late PBO versus referent groups were conducted using two‐sample *t* tests or Wilcoxon rank tests for continuous variables reported as median (interquartile ranges), and chi‐square tests or Fisher exact tests were conducted for categorical variables reported as *n* (%).

Abbreviations: *APOE*, apolipoprotein E; PBO, premenopausal bilateral oophorectomy.

Study findings that were statistically significant were bolded.

^a^ Models are adjusted for age.

^b^ Chi‐square test comparing married/living as married to other categories.

^c^ Chi‐square test comparing all four categories.

^d^ Chi‐square test comparing White versus non‐White.

Linear regression models were used to compare imaging biomarkers of AD in early and late PBO groups to the referent group after adjusting for age at imaging assessment or for both age at imaging assessment and *APOE* ε4 status. Women who underwent early PBO had smaller entorhinal cortex thickness compared to the referent women after adjusting for age (PBO *p* = 0.018), or for age and *APOE* ε4 status (PBO *p* = 0.022). Women who underwent early PBO also showed a trend of greater tau deposition compared to the referent women after adjusting for age (PBO *p* = 0.13), or for age and *APOE* ε4 status (PBO *p* = 0.16), but this difference did not reach statistical significance (Table 2). No differences in the biomarkers of AD were observed between women with late PBO and the referent women (Table 3).

**TABLE 2:** Early PBO and imaging biomarkers of AD.

		Main effect only					Interaction models			
Imaging					*APOE* unadj.					*APOE* unadj.
Outcome	Predictor	Estimate	SE	*p* value	*p* value	Predictor	Estimate	SE	*p* value	*p* value
Log PiB SUVR	Age	0.010	0.002	<0.0001	0.0001	Age	0.006	0.003	0.026	0.0278
Aβ load	*APOE*	0.076	0.025	0.0028		*APOE*	0.079	0.025	0.0014	
	PBO	0.017	0.023	0.46	0.39	PBO	−0.057	0.038	0.13	0.21
						Age x PBO	0.013	0.005	**0.014**	**0.026**
Flortaucipir SUVR	Age	0.009	0.002	<0.0001	<0.0001	Age	0.008	0.003	0.0026	0.0020
Tau load	*APOE*	0.030	0.023	0.20		*APOE*	0.031	0.023	0.18	
	PBO	0.031	0.022	0.16	0.13	PBO	0.006	0.036	0.87	0.78
						Age x PBO	0.005	0.005	0.37	0.41
Temporal lobe	Age	−0.017	0.006	0.0066	0.0059	Age	−0.009	0.007	0.20	0.24
Cortex thickness	*APOE*	0.058	0.064	0.36		*APOE*	0.046	0.063	0.47	
	PBO	−0.070	0.058	0.23	0.28	PBO	0.084	0.096	0.38	0.24
						Age x PBO	−0.027	0.014	**0.045**	**0.022**
Entorhinal cortex	Age	−0.009	0.006	0.15	0.16	Age	−0.006	0.007	0.42	0.49
Thickness	*APOE*	0.047	0.065	0.47		*APOE*	0.042	0.065	0.52	
	PBO	−0.136	0.060	**0.022**	**0.0183**	PBO	−0.074	0.099	0.46	0.51
						Age x PBO	−0.011	0.014	0.43	0.35

*Note*: Linear regression models testing the association of Aβ (log PiB SUVR), tau (flortaucipir SUVR), and neurodegeneration (entorhinal cortex thickness and temporal lobe cortex thickness) imaging biomarkers of Alzheimer's disease with early PBO were adjusted for age at assessment. *APOE* ε4 status adjusted and unadjusted (unadj. *APOE*) models, and models showing the interaction with age are included.

Abbreviations: Aβ, amyloid beta; AD, Alzheimer's disease; *APOE*, apolipoprotein E; PBO, premenopausal bilateral oophorectomy; PiB, Pittsburgh compound B; SUVR, standardized uptake value ratio; unadj., unadjusted.

Study findings that were statistically significant were bolded.

**TABLE 3:** Late PBO and imaging biomarkers of AD.

		Main effect only					Interaction models			
Imaging					*APOE* unadj.					*APOE* unadj.
Outcome	Predictor	Estimate	SE	*p* value	*p* value	Predictor	Estimate	SE	*p* value	*p* value
Log PiB SUVR	Age	0.005	0.002	0.0226	0.030	Age	0.006	0.003	0.0275	0.033
Aβ load	*APOE*	0.118	0.025	<0.0001		*APOE*	0.118	0.025	<0.0001	
	PBO	0.023	0.022	0.30	0.32	PBO	0.046	0.042	0.28	0.28
						Age x PBO	−0.003	0.005	0.53	0.51
Flortaucipir SUVR	Age	0.007	0.002	<0.0001	<0.0001	Age	0.008	0.002	0.0003	0.0002
Tau load	*APOE*	0.041	0.020	0.0375		*APOE*	0.041	0.020	0.0383	
	PBO	0.008	0.018	0.67	0.72	PBO	0.020	0.035	0.54	0.54
						Age x PBO	−0.002	0.004	0.65	0.62
Temporal lobe	Age	−0.013	0.006	0.0194	0.022	Age	−0.009	0.007	0.20	0.45
Cortex thickness	*APOE*	0.017	0.064	0.79		*APOE*	0.013	0.064	0.84	
	PBO	−0.019	0.059	0.75	0.67	PBO	0.087	0.111	0.43	0.22
						Age x PBO	−0.014	0.012	0.26	0.25
Entorhinal cortex	Age	−0.009	0.006	0.14	0.16	Age	−0.005	0.007	0.46	0.48
Thickness	*APOE*	−0.104	0.067	0.84		*APOE*	−0.017	0.067	0.80	
	PBO	−0.014	0.061	0.82	0.74	PBO	0.063	0.116	0.59	0.61
						Age x PBO	−0.010	0.013	0.43	0.42

*Note*: Linear regression models testing the association of Aβ (log PiB SUVR), tau (flortaucipir SUVR), and neurodegeneration (entorhinal cortex thickness and temporal lobe cortex thickness) imaging biomarkers of Alzheimer's disease with late PBO were adjusted for age at assessment. *APOE* ε4 status adjusted and unadjusted (unadj. *APOE*) models, and models showing the interaction with age are included.

Abbreviations: Aβ, amyloid beta; *APOE*, apolipoprotein E; PBO, premenopausal bilateral oophorectomy; PiB, Pittsburgh compound B; SUVR, standardized uptake value ratio; unadj., unadjusted.

**FIGURE 2:**
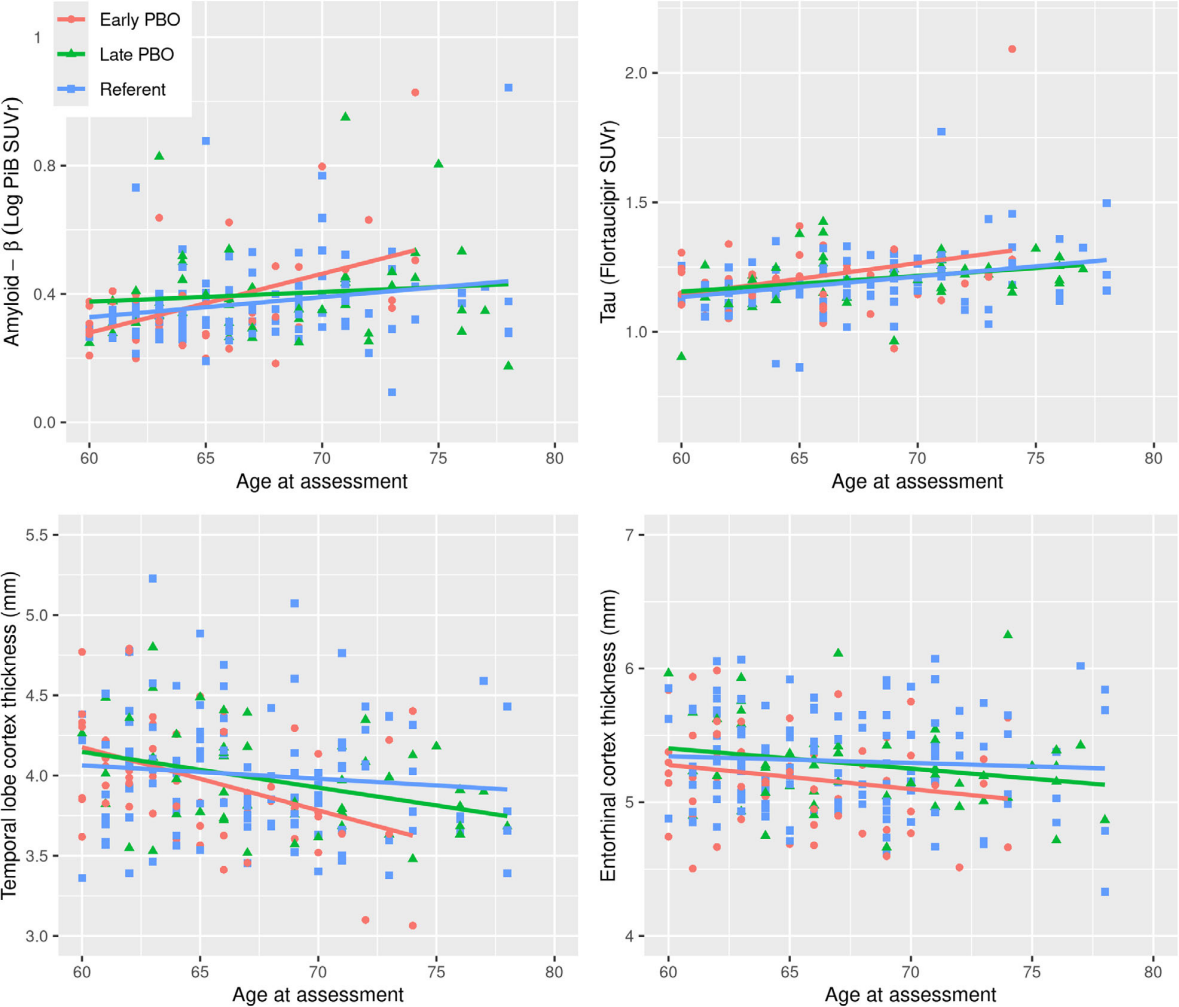
Imaging biomarkers of Alzheimer's disease by age and premenopausal bilateral oophorectomy status: The Aβ (log PiB SUVR), tau (flortaucipir SUVR), and neurodegeneration (entorhinal cortex thickness and temporal lobe cortex thickness) imaging biomarkers of Alzheimer's disease by age are displayed as points and regression lines. Regression lines are colored in red for women who underwent PBO before the age of 46 years (early PBO), colored in green for women who underwent PBO between the ages 46 and 49 years (late PBO), and colored in blue for referent women who did not undergo PBO. The early PBO group had higher Aβ and lower temporal lobe cortical thickness with increasing age compared to the referent group. Although entorhinal cortex thickness was lower in early PBO compared to the referent group, age did not influence this difference. By contrast, imaging biomarkers of Alzheimer's disease in the late PBO group were not different from the referent group. Aβ, amyloid beta; PBO, premenopausal bilateral oophorectomy; PiB, Pittsburgh compound B; SUVR, standardized uptake volume ratio.

In additional models, we examined the interaction between PBO (early and late) and age at assessment in relation to the imaging biomarkers of AD. There was an interaction between PBO and age such that women with early PBO had a greater deposition of Aβ compared to the referent women with increasing age at assessment (age by PBO interaction *p* = 0.026). The interaction persisted also after adjusting for *APOE* ε4 status (age by PBO interaction *p* = 0.014). Furthermore, women with early PBO had an AD‐associated pattern of smaller temporal lobe cortical thickness compared to the referent women when assessed at an older age (age by PBO interaction *p* = 0.022). The interaction persisted also after adjusting for *APOE* ε4 status (age by PBO interaction *p* = 0.045; Table 2). These effects were not found in women who underwent late PBO (Table 3). Figure 2 shows the Aβ (log PiB SUVR), tau (flortaucipir SUVR), and neurodegeneration (entorhinal cortex thickness and temporal lobe cortex thickness) imaging biomarkers of AD by age at imaging assessment in the early PBO, late PBO, and referent groups.

Furthermore, we assessed whether Aβ levels (PiB SUVR) modified the association between PBO and tau (flortaucipir SUVR). Women who underwent early PBO had higher tau levels compared to the referent women after adjusting for age and Aβ levels (early PBO *p* = 0.047), or for age, Aβ levels, and *APOE* ε4 status (early PBO *p* = 0.049). In the interaction models, we found that higher Aβ (PiB SUVR) modified the association of tau (flortaucipir SUVR) with early PBO (Table 4).

**TABLE 4 alz14469-tbl-0004:** Linear regression models testing the association of tau (flortaucipir SUVR), with early PBO were adjusted for age at assessment.

		Main effect only				Interaction models				
	Predictor	Estimate	SE	*p* value	*APOE* unadj. *p* value	Predictor	Estimate	SE	*p* value	*APOE* unadj. *p* value
**Early PBO** **versus referent**										
Flortaucipir SUVR	Age	0.005	0.002	0.010	0.008	Age	0.005	0.002	0.016	0.013
Tau load	*APOE*	−0.016	0.021	0.436		*APOE*	−0.004	0.021	0.838	
	Aβ	0.195	0.038	<0.0001	<0.0001	Aβ	0.116	0.047	0.014	0.012
	PBO	0.038	0.019	**0.049**	**0.047**	PBO	−0.250	0.107	0.021	0.016
						Aβ x PBO	0.194	0.071	**0.007**	**0.005**
**Late PBO** **versus referent**										
Flortaucipir SUVR	Age	0.005	0.002	0.001	0.001	Age	0.005	0.002	0.001	0.001
Tau load	*APOE*	−0.002	0.019	0.935		*APOE*	−0.001	0.019	0.945	
	Aβ	0.117	0.034	0.0007	0.0004	Aβ	0.113	0.040	0.006	0.004
	PBO	0.012	0.016	0.436	0.420	PBO	−0.010	0.103	0.924	0.935
						Aβ x PBO	0.015	0.068	0.828	0.836

*Note APOE*: ε4 status adjusted and unadjusted (unadj. *APOE*) models, and models showing the interaction with Aβ (log PiB SUVR) are included.

Abbreviations: Aβ, amyloid beta; *APOE*, apolipoprotein E; PBO, premenopausal bilateral oophorectomy; PiB, Pittsburgh compound B; SUVR, standardized uptake value ratio; unadj., unadjusted.

Study findings that were statistically significant were bolded.

Finally, we investigated the association of Aβ (PiB SUVR) and tau (flortaucipir SUVR) levels with cognitive performance (the 3MS scores) in each group. We found that higher tau levels (flortaucipir SUVR) were associated with lower 3MS scores in the early PBO group (*β* = −0.011; *p* = 0.003), but not in the late PBO group (*β* = −0.003; *p* = 0.44) or the referent group (*β* = −0.001; *p* = 0.68) after adjusting for age. These findings did not change after adjusting for both age and *APOE* ε4 status in the early PBO group (*p* = 0.002), late PBO group (*p* = 0.43), and the referent group (*p* = 0.86).

Because most of the participants who underwent PBO were using HT (*n* = 40; 65.6% in the early PBO and *n* = 37; 77.1% in the late PBO), we performed a sensitivity analysis excluding women who did not use HT through the age of 50 years. The overall trends and estimates in these sensitivity analyses were comparable to the entire cohort (Tables S1 and S2 in supporting information).

## 4 DISCUSSION

In this cross‐sectional study, women who underwent PBO before the age of 46 years had more AD‐related neuroimaging abnormalities, which increased with age. Women who underwent PBO before the age of 46 also had greater tau load at higher Aβ levels compared to women who did not undergo PBO. These effects were not observed in women who underwent PBO at the age of 46 to 49 years (i.e., late PBO), perhaps because they reached menopause within the age range of spontaneous menopause. Of the AD imaging biomarkers that we examined, both Aβ and the AD‐like pattern of neurodegeneration showed abnormalities in women with early PBO. These biomarkers included an age‐dependent elevation in Aβ and a reduction in AD‐like pattern of temporal lobe cortical thickness in the early PBO group compared to the referent group. A smaller entorhinal cortical thickness was also present in the early PBO group compared to the referent group, but this observation was independent of age at assessment and was consistently observed over the age of 60 years.

There is compelling evidence that endogenous ovarian hormones, particularly estrogens, may modify Aβ pathology. Estrogens reduce inflammatory responses,^21^ especially to Aβ,^22^ improve cerebrospinal fluid clearance of insoluble Aβ,^23^ and increase the expression of non‐toxic soluble Aβ.^24^ In addition, estrogens have a regulatory role in AD Aβ precursor protein.^25^ Transdermal 17β‐estradiol given to recently postmenopausal women was associated with lower levels of Aβ, particularly in women who were carriers of *APOE* ε4 in a randomized clinical trial.^5^ Our findings from the MOA‐2 cohort confirmed that PBO before the age of 46 years is associated with elevated Aβ levels later in life, but the association varies depending on the age at assessment. Although the increase in Aβ with age was greater in the early PBO group compared to the referent group, this finding did not change after adjusting for *APOE* ε4 status and was not modified by *APOE* ε4 status when tested in interaction models.

Although the tau PET biomarker showed a trend of higher tau deposition in early PBO compared to the referent group, this difference was not statistically significant. This finding did not change after adjusting for *APOE* ε4 status and was not modified by *APOE* ε4 status when tested in interaction models. However, when we investigated the influence of Aβ on tau deposition in the early PBO compared to the referent group, we found that tau levels were higher in the early PBO group compared to the referent group at higher Aβ levels. Furthermore, higher tau levels were associated with lower cognitive performance measured with the 3MS only in the early PBO group, but not the late PBO or the referent groups. Because the increase in Aβ precedes the increase in tau measured on PET and the decline in cognitive function during the evolution of AD,^26^ it is possible that the PBO cohort that we studied was relatively young to show effects of PBO on tau deposition, particularly in the early PBO group (median age at assessment of 64.8 years).^27^ Therefore, we could only observe elevated tau levels in women who already had greater Aβ in the early PBO compared to the referent group, and tau was associated with cognitive performance only in the early PBO group. Consistent with our findings, a recent study indicated that earlier age at menopause was associated with higher regional tau in women with high Aβ levels; however, surgical and spontaneous menopause were combined in this study.^28^ It is possible that there are differential effects of spontaneous and surgical menopause on both Aβ and tau deposition.

The MRI biomarkers of neurodegeneration that we studied included the entorhinal cortical thickness, which is the earliest cortical region to be affected by AD,^19^ and temporal cortical thickness, a biomarker of neurodegeneration associated with AD dementia.^17^ Neurodegenerative changes due to bilateral ovariectomy have been demonstrated in transgenic animal models of AD using imaging.^29^, ^30^ Furthermore, greater estrogen exposure such as premenopausal status, longer reproductive span, higher number of children, and use of hormonal contraceptives and menopausal hormone therapies were associated with larger gray matter volumes in women in midlife, and the findings were independent of the *APOE* ε4 status.^31^ Consistent with these prior studies, we found that the abrupt cessation of ovarian hormones in women who underwent early PBO compared to the referent group was associated with lower temporal lobe cortical thickness at older ages, which was independent of *APOE* ε4 status. This finding is also consistent with the Aβ levels showing abnormalities at older ages in our study, again independent of *APOE* ε4 status.

Women who underwent early PBO had smaller entorhinal cortex thickness compared to the referent women, and this finding was not modified by age at imaging assessment or by *APOE* ε4 status. Involvement of the medial temporal lobe structures has been previously demonstrated in a smaller cohort of women who underwent PBO; however, the differential involvement in early PBO versus late PBO was not investigated.^32^ Furthermore, in middle‐aged women who underwent PBO, the volume of the hippocampus, which has connections to the entorhinal cortex, was lower in those who did not use hormone therapy after PBO than controls in a recent Canadian study.^33^ Our study showed that the neurodegeneration in the entorhinal cortex is present as early as at age 60 years in women who underwent PBO before the age of 46 years. Our findings combined with the results from previous studies suggest that the medial temporal lobe is particularly vulnerable to the abrupt loss of ovarian hormones caused by early PBO.[Table alz14469-tbl-0004]

Hormone therapies after spontaneous menopause may influence Aβ levels.^5^ However, when we excluded women who did not use hormone therapies after PBO and through the age of 50 years, the results remained similar to the entire cohort, suggesting that hormone therapies did not influence imaging biomarker outcomes. This finding needs to be interpreted with caution because MOA‐2 included a cohort of women who underwent PBO from 1988 through 2007 and these women were treated with historical hormone therapies (primarily oral conjugated equine estrogen) rather than with current hormone therapies. Furthermore, women with menopausal vasomotor symptoms, sleep disturbances, and cognitive complaints would likely have been prescribed hormone therapy more often than women who did not present with these symptoms,^34^ and these symptoms are associated with poor imaging and AD biofluid biomarker outcomes later in life.^35^, ^36^ Thus, the influence of hormone therapy on PBO outcomes warrants further assessment in a clinical trial.

Although this study has several strengths including the evaluation of a relatively large well‐characterized, epidemiologic cohort with a complete panel of imaging biomarkers of AD, and the classification of PBO as early and late PBO, there are also several limitations. First, it was not possible to effectively investigate the hormone therapy effects on the AD imaging biomarkers later in life. Second, the imaging biomarker abnormalities of Aβ and AD‐like neurodegeneration were only observed with increasing age at assessment. Therefore, we hypothesize that the imaging biomarkers would have been abnormal across the entire age range among women at older ages. Finally, investigating the imaging biomarkers in a longitudinal study with repeated imaging over time would have more power to determine the change in AD imaging biomarker abnormalities with aging in early PBO, and might be less susceptible to sampling biases.

In conclusion, PBO before the age of 46 years is associated with entorhinal cortex thinning and elevated tau at higher levels of Aβ load at all ages of assessment, and with an increased Aβ load along with an AD‐like pattern of atrophy at older ages at assessment. Longitudinal follow‐up with serial imaging of women who underwent PBO before the age of 46 years may reveal the evolution of AD in these women, which is critical for preventive strategies. These findings provide critical insights guiding the health‐care professionals and the women considering PBO prior to the age of 46 years for non‐malignant indications and outside of the setting of high genetic risk of ovarian cancer.

## CONFLICT OF INTEREST STATEMENT

Dr. Kantarci was partially funded by the Kathrine B. Andersen Professorship of Women's Health Research of the Mayo Clinic. Dr. Kantarci consulted for Biogen. She received research support form Eli Lilly. Dr. Kapoor has been a consultant for Astellas and Mithra Pharmaceuticals, Scynexis, and Womaness. She receives grant support form Mithra Pharmaceuticals. She has received payment for development of educational content from Med Learning Group and Academy of Continued Healthcare Learning. She has received honoraria for CME activity from PriMed and OBG Management. Dr. Rocca was partially funded by the Ralph S. and Beverley E. Caulkins Professorship of Neurodegenerative Diseases Research of the Mayo Clinic. Dr. Mielke has served on scientific advisory boards and/or has consulted for Biogen, LabCorp, Lilly, Merck, PeerView Institute, Siemens Healthineers, and Sunbird Bio unrelated to the current manuscript. The other authors declare no competing interests. Author disclosures are available in the supporting information.

## CONSENT STATEMENT

All human subjects provided informed consent or confirmed that consent was not necessary.

## Supporting information



Supporting Information

Supporting Information
