# Supplementary material for: Premenopausal bilateral oophorectomy and Alzheimer's disease imaging biomarkers later in life
Source: Alzheimers Dement. 2024 Dec 23;21(7):e14469. doi: 10.1002/alz.14469 (PMC12226247; doi:10.1002/alz.14469)
Supplement: Supplementary file 1 — Supporting Information [file ALZ-21-e14469-s002.docx]

**Supplemental Table 1 in women who underwent early PBO and took hormone therapy until 50 years of age (n=159; early PBO n=40, referent n=119).** Linear regression models testing the association of amyloid-β (log PiB-SUVr), tau (Flortaucipir SUVr), and neurodegeneration (entorhinal cortex thickness and temporal lobe cortical thickness) imaging biomarkers of Alzheimer’s disease with early PBO were adjusted for age at assessment.

|  | **Main Effect-only** | | | |  | **Interaction Models** | | | |
| --- | --- | --- | --- | --- | --- | --- | --- | --- | --- |
| Imaging outcome | Predictor | Estimate | SE | P-value |  | Predictor | Estimate | SE | P-value |
| Log PiB-SUVr | Age | 0.009 | 0.003 | 0.0003 |  | Age | 0.006 | 0.003 | 0.0253 |
| Amyloid-β load | PBO | 0.032 | 0.027 | 0.2264 |  | PBO | -0.045 | 0.045 | 0.3141 |
|  |  |  |  |  |  | Age*PBO | 0.013 | 0.006 | 0.0334 |
| Flortaucipir SUVr | Age | 0.010 | 0.002 | <.0001 |  | Age | 0.008 | 0.003 | 0.0019 |
| Tau load | PBO | 0.055 | 0.026 | 0.0327 |  | PBO | 0.005 | 0.042 | 0.9145 |
|  |  |  |  |  |  | Age* PBO | 0.009 | 0.006 | 0.1304 |
| Temporal Lobe | Age | -0.013 | 0.006 | 0.0351 |  | Age | -0.008 | 0.007 | 0.2363 |
| Cortex Thickness | PBO | -0.087 | 0.066 | 0.1830 |  | PBO | 0.047 | 0.113 | 0.6749 |
|  |  |  |  |  |  | Age* PBO | -0.022 | 0.015 | 0.1455 |
| Entorhinal Cortex | Age | -0.009 | 0.007 | 0.1799 |  | Age | -0.005 | 0.007 | 0.4951 |
| Thickness | PBO | -0.090 | 0.068 | 0.1885 |  | PBO | 0.014 | 0.119 | 0.9041 |
|  |  |  |  |  |  | Age* PBO | -0.017 | 0.016 | 0.2848 |

**Supplemental Table 2 in women who underwent late PBO and took hormone therapy until 50 years of age (n=156, late PBO n=37, referent n=119)**. Linear regression models testing the association of amyloid-β (log PiB-SUVr), tau (Flortaucipir SUVr), and neurodegeneration (entorhinal cortex thickness and temporal lobe cortex thickness) imaging biomarkers of Alzheimer’s Disease with late PBO were adjusted for age at assessment.

|  | **Main Effect-only** | | | |  | **Interaction Models** | | | |
| --- | --- | --- | --- | --- | --- | --- | --- | --- | --- |
| Imaging outcome | Predictor | Estimate | SE | P-value |  | Predictor | Estimate | SE | P-value |
| Log PiB-SUVr | Age | 0.005 | 0.002 | 0.0280 |  | Age | 0.006 | 0.003 | 0.0308 |
| Amyloid-β load | PBO | 0.022 | 0.026 | 0.3896 |  | PBO | 0.049 | 0.053 | 0.3556 |
|  |  |  |  |  |  | Age*PBO | -0.003 | 0.005 | 0.5660 |
| Flortaucipir SUVr | Age | 0.007 | 0.002 | 0.0001 |  | Age | 0.008 | 0.002 | 0.0001 |
| Tau load | PBO | 0.003 | 0.019 | 0.8623 |  | PBO | 0.038 | 0.040 | 0.3345 |
|  |  |  |  |  |  | Age*PBO | -0.004 | 0.004 | 0.3142 |
| Temporal Lobe | Age | -0.010 | 0.006 | 0.0750 |  | Age | -0.008 | 0.007 | 0.2218 |
| Cortex Thickness | PBO | -0.054 | 0.064 | 0.4005 |  | PBO | 0.011 | 0.130 | 0.9322 |
|  |  |  |  |  |  | Age*PBO | -0.008 | 0.013 | 0.5674 |
| Entorhinal Cortex | Age | -0.004 | 0.006 | 0.4678 |  | Age | -0.005 | 0.007 | 0.4815 |
| Thickness | PBO | -0.061 | 0.067 | 0.3607 |  | PBO | -0.080 | 0.136 | 0.5552 |
|  |  |  |  |  |  | Age*PBO | 0.002 | 0.014 | 0.8712 |
